# The Prevalence of Iron Deficiency in Atrial Fibrillation: Low Hanging Fruit?

**DOI:** 10.3390/medicina58101492

**Published:** 2022-10-19

**Authors:** Bachar Alabdullah, David Ferreira, Erin Bourke, Harish Kamalanathan, Ibrahim Elashri, Kushal Porwal, Michael J. Tiller, Payal H. Gadre, Sarah Jones, Michael McGee

**Affiliations:** 1 Cardiology Department, Tamworth Rural Referral Hospital, Dean Street, North Tamworth, NSW 2340, Australia; 2 Cardiovascular Department, John Hunter Hospital, New Lambton Heights, Newcastle, NSW 2305, Australia

**Keywords:** atrial fibrillation, iron deficiency, epidemiology

## Abstract

*Background and Objectives*: Atrial fibrillation (AF) is the most common sustained arrhythmia worldwide. The relationship between AF and iron deficiency is poorly understood. *Materials and Methods*: We conducted an observational study investigating the prevalence of iron deficiency in those with AF. Iron deficiency was defined by the American College of Cardiology (ACC) criteria for iron deficiency in heart failure. *Results*: Of 134 eligible subjects, 81 (60.4%) met the ACC definition of iron deficiency in heart failure. Those who were iron deficient were more likely to be female (OR 1.876, *p* = 0.005), have a history of diabetes mellitus (OR 3.085, *p* = 0.001) a history of stroke (OR 3.147, *p* = 0.016), and have higher CHA_2_DS_2_-VASc (*p* ≤ 0.0001) and Charlson Comorbidity Index scores (CCI) (*p* = 0.007). *Conclusions*: The prevalence of iron deficiency in those with AF appears high and warrants evaluation in a prospective study.

## 1. Introduction

Atrial fibrillation (AF) is the most common sustained arrhythmia worldwide [1]. The relationship between AF and iron deficiency is poorly understood. Iron deficiency occurs in chronic diseases in which the inflammatory state leads to reduced iron absorption and utilisation [2,3]. In those with heart failure, intravenous iron has been shown to reduce the composite endpoint of heart failure hospitalisation and cardiovascular mortality [4,5].

Likewise, AF involves a chronic inflammatory state which may contribute to iron deficiency [6]. Elevated levels of pro-inflammatory cytokines including C-reactive protein and tumor necrosis factor-α predict an increased risk of atrial fibrillation [7]. Moreover, systemic inflammation from illness and surgical intervention also increases arrhythmic risk [8]. There are mechanistic studies which link iron deficiency with electrophysiological changes which predispose to symptomatic arrhythmia [9,10,11]. However, the association between iron deficiency and AF has not yet been adequately assessed. Iron deficiency may represent a low risk and cheap therapeutic target for those with atrial fibrillation.

We performed a retrospective observational study to explore the relationship between AF and iron deficiency. We also aimed to assess if iron deficiency in AF is associated with worse outcomes.

## 2. Materials and Methods

Medical records of patients with a diagnosis of AF receiving care through either John Hunter Hospital (Newcastle, NSW, Australia) or Tamworth Rural Referral Hospital (Tamworth, NSW, Australia) were reviewed. Inclusion criteria were age of 18 years or over and hospital presentation between the 1st of January 2015 and 30th of April 2020, AF diagnosis as an active issue, and iron studies 6 months before or after the index presentation. Ethics approval was obtained for this study through the Hunter Research Ethics Committee.

The American College of Cardiology’s (ACC) definition of iron deficiency in heart failure (Ferritin <100 µg/L or ferritin of 100–299 µg/L with a transferrin saturation <20%) was used [12]. Data from eligible digital medical records were collected including demographics, date and time, admission date and time, discharge date and time, medical comorbidities, regular medications at time of presentation, echocardiographic findings, and serology results. Chronic kidney disease was defined as an estimated Glomerular Filtration Rate (GFR) of <60 mL/min or 2 different blood samples at least 3 months apart.

Patient outcomes were recorded through review of digital medical records including hospital representations, cardiovascular outcomes (myocardial infarctions (MI), transient ischaemic attacks (TIA), and ischaemic stroke), and end of life events. The data collected were then used to calculate each subject’s length of stay (LOS), CHA_2_DS_2_-VASc score, HAS-BLED score, Charlson Comorbidity Index (CCI), and time to outcome after discharge.

Statistical analysis was performed IBM SPSS Statistics version 23.0.0.0. Subjects were divided into two groups: iron deficient and non-iron deficient. A chi-squared test with an alpha of 0.05 was used to compare nominal indices between the groups. For scale indices, an independent-samples T test was used to compare the means between the two groups.

The primary endpoint of this study was the proportion of patients with AF who have iron deficiency as defined by the ACC. Secondary endpoints included rates of hospital representation and readmission (related to AF or otherwise), length of stay, CHA_2_DS_2_-VASc score, HAS-BLED score, CCI, and mortality.

## 3. Results

**Figure 1:**
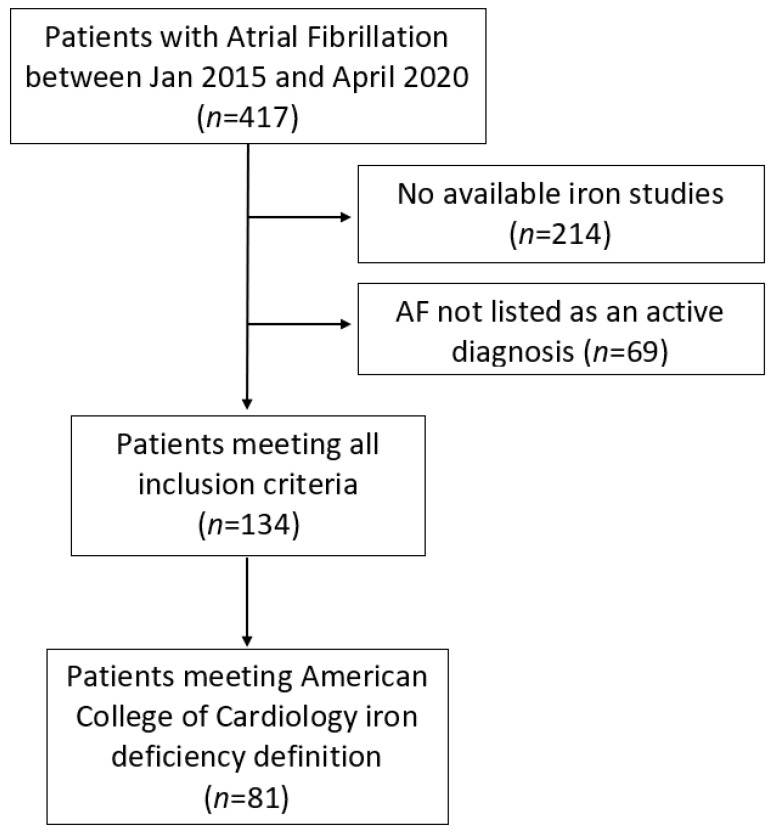
Flow diagram of patient inclusion.

134 patients met inclusion criteria for this study (see Figure 1). 76 (56.7%) were male and 58 (43.3%) were female. Baseline demographics are listed in Table 1. Of 134 subjects, 81 (60.4%) met the ACC definition of iron deficiency. Average follow-up for the cohort was 3.5 ± 1.6 years. During follow-up, 110 (82.1%) presented to hospital, of which 45 (38.5%) were due to AF recurrence and 24 (22.0%) due to an end-of-life event.

Those who met the ACC definition of iron deficiency were more likely to be female (OR 1.9 [1.2–3.0 95% CI], *p* = 0.005), have a history of diabetes mellitus (OR 3.1 [1.5–6.5 95% CI], *p* = 0.001) and a history of stroke (OR 3.1 [1.1–8.7 95% CI]), *p* = 0.016) (Supplementary Table S2). The iron deficiency group showed significantly higher CHA_2_DS_2_-VASc (4.2 vs. 2.9 *p* ≤ 0.0001) and CCI scores (6.1 vs. 4.8, *p* = 0.007) (Table 2).

There were no differences in hospital presentation or readmission. Iron deficiency did not associate with MI (OR 1.1 [0.18–6.06 95% CI], *p* = 1.0) or ischaemic cerebrovascular events (OR 1.4 [0.27–7.35 95% CI], *p* = 1.000), but did trend towards increased risk of mortality (OR 2.1 [0.91–4.9 95% CI], *p* = 0.067) (Supplementary Table S2).

## 4. Discussion

In this retrospective observational study of patients who presented with AF, the prevalence of iron deficiency based on the ACC heart failure criteria was 60%, irrespective of anaemia status. The rates of iron deficiency in our study are similar to other reports [13]. We used the ACC’s definition of iron deficiency for heart failure in our analysis. Similar to heart failure, chronic inflammation is observed in AF and has previously been proposed as the possible mechanism of iron deficiency [6]. Adamsson et al. also inferred this by proposing chronic inflammation as a possible mechanism leading to increased red cell distribution width (RDW), which they showed was associated with AF [10].

Females were more likely to be iron deficient compared to males. This is expected, reflecting the differential incidence of iron deficiency in the general population between the two sexes. Both the CHA_2_DS_2_-VASc and CCI scores were higher in iron deficient subjects. Having a higher CHA_2_DS_2_-VASc score increases the likelihood of the use of anticoagulation therapy, which may increase subclinical gastrointestinal blood loss and possible iron deficiency.

Secondary endpoint analysis showed a large burden of comorbid illnesses. This is demonstrated through a mean CCI of 5.6, reflecting a 10-year survival of less than 21%. When comparing the iron deficient versus non-deficient groups, the CCI was higher on average in those with iron deficiency (*p* = 0.007). Underlying comorbidities are likely to confound the association between AF and iron deficiency, however the theoretical benefits of iron replacement in this population are still of great interest.

Limitations of the study include its retrospective nature, the small sample size, and the lack of a formal comparison between the incidence of iron deficiency in patients with AF against those without. Multivariate analysis was not performed, and there was a high prevalence of confounding comorbidities such as congestive cardiac failure (35.3%) and chronic kidney disease (33.8%), both of which have an established effect on iron metabolism [14]. Moreover, the incidence of iron deficiency in our study may be artificially high because of selection bias. Those referred for iron studies are more likely to have a separate indication for testing, for example, concern over bleeding or symptoms suggestive of iron deficiency. Without a comparator group, the association between atrial fibrillation and iron in this study is hypothesis-generating only. Iron level cut-offs used for heart failure have not been validated in atrial fibrillation. The treatment provided for iron deficiency was not assessed as data from the primary care physician were not available.

## 5. Conclusions

In this study, we saw a hypothesis-generating link between AF and iron deficiency. Further prospective data are required to characterise this relationship. If confirmed, the benefits of iron replacement in symptomatic AF should be investigated, as it may prove to be a potentially beneficial therapeutic target, regardless of underlying confounding comorbidities.

## Figures and Tables

**Table 1:** Baseline Clinical, Echocardiographic and Serological Characteristics.

Baseline Characteristics	
Age	73.3 (±11.5)
Gender (Male:Female)	76:58
Body Mass Index	30.0 (±6.6)
Chads-Vasc Score	3.7 (±1.8)
HAS-BLED Score	2.8 (±1.2)
Charlson Comorbidity Index	5.6 (±2.6)
Comorbidities	
Hypertension % (*n*)	53.4% (72)
Heart Failure % (*n*)	35.1% (47)
Ischaemic Heart Disease % (*n*)	26.9% (36)
Dyslipidemia % (*n*)	28.4% (38)
Diabetes Mellitus % (*n*)	29.9% (40)
Chronic Kidney Disease % (*n*)	33.6% (45)
Prior Ischemic Stroke/TIA % (*n*)	23.1% (31)
Echocardiographic Characteristics	
LV Ejection Fraction	49.7 (±15.6)
LV Wall Thickness (mm)	11.9 (±2.4)
Left Atrial Area (cm^2^)	28.8 (±9.3)
Serological Characteristics	
Haemoglobin (g/L)	122 (±25.4)
Creatinine (umol/L)	130 (±142)
Ferritin (ug/L)	240 (±288)
Transferrin (g/L)	2.9 (±6.2)
Transferrin Saturation (%)	16.7 (±11.0)

± denotes standard deviation.

**Table 2:** Comparison of iron deficient and non-deficient populations.

Variable	Iron Deficient Group	Iron Non-Deficient Group	*p*-Value
Baseline demographics and clinical characteristics			
Age (years)	74.3 (±1.3)	71.95 (±1.5)	0.258
CHA_2_DS_2_-VASc Score	4.2 (±0.20)	2.9 (±0.24)	<0.0001
HAS-BLED Score	3.0 (±0.14)	2.6 (±0.16)	0.068
CCI	6.1 (±0.30)	4.8 (±0.31)	0.007
Hypertension % (*n*)	55.5% (45)	50.1% (27)	0.60
Heart Failure % (*n*)	39.5% (32)	28.3% (15)	0.18
Ischaemic Heart Disease % (*n*)	28.4% (23)	24.5% (13)	0.62
Diabetes Mellitus % (*n*)	40.7% (33)	13.2% (7)	0.0006
Chronic Kidney Disease % (*n*)	35.8% (29)	30.2% (16)	0.50
Prior Ischemic Stroke/TIA % (*n*)	32.1% (26)	11.3% (6)	0.006
History of Major Bleeding % (*n*)	11.1% (9)	5.6% (3)	0.36
Oral Anticoagulation % (*n*)	54.2% (44)	39.6% (21)	0.096
Anaemia % (*n*)	55.5% (45)	41.5% (22)	0.11
Length of Stay (Days)	11.5 (±1.8)	18.6 (±6.6)	0.297
Readmission at Follow-up % (*n*)	80% (65)	85% (45)	0.49
1-year Mortality % (*n*)	7.4% (6)	9.4% (5)	0.68
Echocardiographic characteristics			
Ejection fraction (%)	48.7 (±2.2)	51.0 (±2.8)	0.506
LV Wall Thickness (mm)	11.7 (±2.7)	12.2 (±1.8)	0.422
LA area (cm^2^)	28.5 (±1.)	29.3 (±2.4)	0.778
Serological results			
Haemoglobin (g/L)	118.0 (±2.6)	128.4 (±3.8)	0.022
Ferritin (ug/L)	98.3 (±8.6)	457.0 (±48.9)	<0.0001
Transferrin Saturation (%)	11.9 (±0.8)	23.9 (±1.7)	<0.0001

CCI, Charlson Comorbidity Index. LV, left ventricle. LA, left atrium. ±, Standard Error.

## Data Availability

Data available on reasonable request from reputable institutions.

## Supplementary Materials

The following supporting information can be downloaded from: https://www.mdpi.com/article/10.3390/medicina58101492/s1, Supplementary Table S1. Frequencies of comorbidities and odds ratio of comorbidities if iron deficient; Supplementary Table S2. Frequencies and odds ratios of demographics and outcomes observed in the iron deficient group; Supplementary Table S3. Distribution of Chronic Kidney Disease based on KDIGO Guidelines
