# Supplementary material for: The Prevalence of Iron Deficiency in Atrial Fibrillation: Low Hanging Fruit?
Source: Medicina (Kaunas). 2022 Oct 19;58(10):1492. doi: 10.3390/medicina58101492 (PMC9610053; doi:10.3390/medicina58101492)
Supplement: Supplementary file 1 [file medicina-58-01492-s001.zip › medicina-1936699-supplementary.pdf]

Supplementary Appendix.

Table S1. Frequencies of comorbidities and odds ratio of comorbidities if iron deficient.

Comorbidity	Whole population n = 134	Iron deficient group n = 81			
	Frequency	Frequency	OR	95% CI	p-value
Ischaemic heart disease (IHD)	36	23	1.187	0.662 - 2.129	0.562
Peripheral vascular disease (PVD)	10	7	1.606	0.435 - 5.931	0.527
Congestive cardiac failure (CCF)	47	32	1.413	0.852-2.343	0.167
Hypertension	72	45	1.104	0.796 - 1.532	0.548
Valvular heart disease	33	20	1.103	0.561 - 1.876	0.934
Hyperlipidaemia	38	26	1.472	0.817 - 2.651	0.186
Obstructive sleep apnoea (OSA)	11	8	1.835	0.510 - 6.601	0.524
Diabetes mellitus	40	33	3.085	1.474 - 6.455	0.001
Anaemia	67	45	1.390	0.958 - 2.016	0.069
Supra-ventricular tachycardia (SVT)	5	4	2.650	0.305 - 23.062	0.648
Chronic kidney disease (CKD)	45	29	1.201	0.727 - 1.984	0.469
Liver disease	4	2	0.638	0.093 - 4.384	0.642
Depression	17	10	0.893	0.363 - 2.194	0.805
Cigarette smoking	28	17	1.261	0.698 - 2.278	0.436
Solid tumour	26	14	0.749	0.376 - 1.490	0.411
Stroke	23	19	3.147	1.134 - 8.734	0.016
Transient ischaemic attack (TIA)	9	7	2.290	0.494 - 10.606	0.271
Venous thromboembolism (VTE)	3	2	1.284	0.119 - 13.805	1.000
Major bleed	12	9	1.926	0.547 - 6.785	0.294
Chronic obstructive pulmonary disease (COPD)	28	19	1.381	0.677 - 2.820	0.367
Peptic ulcer disease	8	5	1.091	0.272 - 4.373	1.000
Dementia	10	7	1.527	0.413 - 5.644	0.521

Table S2. Frequencies and odds ratios of demographics and outcomes observed in the iron deficient group.

**Outcome**	**Frequency**	**OR**	**95% CI**	**P-value**
Sex (Female)	43	1.876	1.167 - 3.016	0.005
Disposition (Admitted to hospital)	69	0.961	0.842 - 1.096	0.562
Any medication that increase bleeding risk	60	1.147	0.907 - 1.451	0.233
Aspirin	19	0.828	0.465 - 1.476	0.524
Anti-platelets	9	0.830	0.330 – 2.089	0.693
Anti-coagulants	44	1.310	0.894 - 1.917	0.149
Representation to hospital	65	0.945	0.808 – 1.105	0.492
AF recurrence	21	0.703	0.440 - 1.124	0.143
Myocardial infarction (MI)	3	1.055	0.184 - 6.058	1.000
Ischemic Stroke	4	1.406	0.269 - 7.352	1.000
Death	18	2.109	0.909 - 4.894	0.067

Table S3. Distribution of Chronic Kidney Disease based on KDIGO Guidelines (131 total patients with renal function available).

**KDIGO Chronic Kidney Disease Stage**	**Proportion of Patients % (n)**
Group 1: GFR >90	11.5% (15)
Group 2: GFR 60-89	40.5% (53)
Group 3a: GFR 45-59	18.3% (24)
Group 3b: GFR 30-44	17.6% (23)
Group 4: GFR 15-29	8.4% (11)
Group 5: GFR <15	2.3% (3)

**KDIGO Chronic Kidney Disease Stage Based on Iron Deficiency Status**		
**Groups**	**Iron Deficient** (n=79)	**Non-Iron Deficient** (n=52)
Group 1: GFR >90	6.3% (5)	19.2% (10)
Group 2: GFR 60-89	44.3% (35)	34.6% (18)
Group 3a: GFR 45-59	21.5% (17)	13.5% (7)
Group 3b: GFR 30-44	16.5% (13)	19.2% (10)
Group 4: GFR 15-29	10.1% (8)	5.7% (3)
Group 5: GFR <15	1.3% (1)	7.7% (4)
